# Moderate Hypoxia Influences Potassium Outward Currents in Adipose-Derived Stem Cells

**DOI:** 10.1371/journal.pone.0104912

**Published:** 2014-08-12

**Authors:** Mayuri Prasad, Vladimir Zachar, Trine Fink, Cristian Pablo Pennisi

**Affiliations:** Laboratory for Stem Cell Research, Department of Health Science and Technology, Aalborg University, Aalborg, Denmark; Rutgers - New Jersey Medical School, United States of America

## Abstract

Moderate hypoxic preconditioning of adipose-derived stem cells (ASCs) enhances properties such as proliferation and secretion of growth factors, representing a valuable strategy to increase the efficiency of cell-based therapies. In a wide variety of cells potassium (K^+^) channels are key elements involved in the cellular responses to hypoxia, suggesting that ASCs cultured under low oxygen conditions may display altered electrophysiological properties. Here, the effects of moderate hypoxic culture on proliferation, whole-cell currents, and ion channel expression were investigated using human ASCs cultured at 5% and 20% oxygen. Although cell proliferation was greatly enhanced, the dose-dependent growth inhibition by the K^+^ channel blocker tetraethylammonium (TEA) was not significantly affected by hypoxia. Under both normoxic and hypoxic conditions, ASCs displayed outward K^+^ currents composed by Ca^2+^-activated, delayed rectifier, and transient components. Hypoxic culture reduced the slope of the current-voltage curves and caused a negative shift in the voltage activation threshold of the whole-cell currents. However, the TEA-mediated shift of voltage activation threshold was not affected by hypoxia. Semiquantitative real-time RT-PCR revealed that expression of genes encoding for various ion channels subunits related to oxygen sensing and proliferation remained unchanged after hypoxic culture. In conclusion, outward currents are influenced by moderate hypoxia in ASCs through a mechanism that is not likely the result of modulation of TEA-sensitive K^+^ channels.

## Introduction

Within the field of regenerative medicine, a multitude of clinical trials using autologous stem cell transplantation are currently under way [1]. While, for historical reasons, bone marrow-derived stem cells are more frequently used, adipose-derived stem cells (ASCs) are increasingly being recognized as a very strong candidate for clinical trials due to their abundance in the human body and easy harvest via minimally invasive procedures. The ASCs have shown to have pro-angiogenic, anti-inflammatory, and anti-apoptotic properties, representing a novel approach for the treatment of a variety of diseases, such as myocardial infarction, stroke, arthritis, and diabetes [2].

The ongoing and proposed clinical trials include not only transplantation of recently harvested cells, but also expansion, preconditioning and predifferentiation of cells prior to implantation. In this context, it is noteworthy that culture of ASCs in hypoxic conditions alters their properties, both in terms of differentiation, secretion of various growth factors, as well as proliferation (reviewed by Zachar et al.) [3]. Interestingly, various ASC properties may by suppressed or enhanced by modulating the degree of hypoxia to which the cells are exposed. By comparing ASCs cultured at 1%, 5%, and 21% oxygen, we demonstrated that the exposure to oxygen levels of 1% is optimal for promotion of the pro-angiogenic properties of ASC in terms of secretion of vascular endothelial growth factor (VEGF-1), whereas culture at 5% oxygen yields faster proliferation [4], [5]. The beneficial effect of moderate hypoxia on ASC proliferation without loss of multipotentiality has been demonstrated even for longer culture periods of almost two months [6].

When ASCs are cultured in hypoxic conditions where the oxygen concentration is at or below 1%, the observed changes in gene expression can in large part be attributed to the increased activity of the central transcription factor hypoxia inducible factor 1 (HIF-1). However, due to the minimal HIF-1 presence above 2% oxygen [7], it seems reasonable that the altered cell behavior at 5% oxygen involves mechanisms which are independent of HIF-1. Another key cellular mechanism for oxygen sensing comprises ion channels that are responsive to acute as well as to prolonged hypoxia [8]. As studies have shown, hypoxia modulates the expression and/or function of ion channels in a wide variety of cells, including T lymphocytes [9], glomerular podocytes [10], pulmonary smooth muscle cells [11], [12], trophoblast cells [13], neural progenitor cells [14], and pheochromocytoma cells [15], [16]. Although different ion channel families display oxygen sensitivity, K^+^ channels distinctively play an important role in conferring the cellular sensitivity to hypoxia [17].

Human mesenchymal stem cells (MSCs) derived from different sources like adipose tissue, umbilical cord vein and bone marrow express a wide range of ion channels subunits [18]–[20]. These include a plethora of voltage-gated K^+^ channels (such as Kv1.1, Kv1.2, Kv1.4, Kv4.2, and Kv4.3), as well as voltage-gated L-type Ca^2+^ channels (α1C subunit), hyperpolarization activated cyclic nucleotide-gated K^+^ channel 2 (HCN2), large conductance Ca^2+^-activated K^+^ channel (MaxiK), and inwardly-rectifying K^+^ channel (Kir2.1). However, the functional role of most of these channels in MSCs has not been clearly established yet. Studies have demonstrated that MSCs display cell-cycle dependent changes in membrane potential and K^+^ currents, suggesting a key role of K^+^ channels in controlling cell proliferation [21]. In line with these findings, the K^+^ channel blocker tetraethylammonium (TEA) has been shown to inhibit the proliferation of ASCs, although specific K^+^ channel subunits could not be clearly identified [19]. More recently, it has been shown that voltage-gated K^+^ channels and Ca^2+^-activated K^+^ channels play an important role in regulation of MSCs proliferation [22]. In addition to Kv channels, the activity of other ion channels, such as the voltage-gated Ca^2+^ channel, has been correlated with an increase in cell proliferation induced by hypoxia [14]. Thus, the results of these recent studies suggest that the expression and/or activity of ion channels in ASCs may be altered following moderate hypoxic culture.

In this work, we investigated whether ion channels that are expressed by human ASCs are involved in the increased proliferative activity of the cells cultured at 5% oxygen. In particular, we focused on the involvement of the TEA-sensitive K^+^ channels, which underlie the main outward currents that regulate the proliferation of MSCs.

## Materials and Methods

### Cell culture

The human ASC cultures were established from adipose tissue that was obtained during elective liposuction from three healthy donors. The patients gave written informed consent and the clinical protocol was approved by the regional Committee on Biomedical Research Ethics of Northern Jutland, Denmark (project no. VN 2005/54). The generation and characterization of these cell cultures (labelled as ASC12, ASC21 and ASC23) in regards to their capacity to differentiate into multiple lineages have previously been described in detail [4], [23]–[26]. All experiments were conducted on cells at passage 4. Growth medium consisted of 90% α-MEM (Invitrogen, Taastrup, Denmark), 10% fetal calf serum (Helena Bioscience, UK), 100 IU/ml penicillin, 0.1 mg/ml streptomycin, and 0.05 mg/ml gentamicin (all from Invitrogen). Cells were cultured at 37°C and 5% CO_2_, in a standard incubator at ambient oxygen for the normoxic experiments or in a hypoxic workstation (Xvivo System, Biospherix, Lacona, NY) at 5% oxygen for the hypoxic experiments.

### TEA growth inhibition assay

Cells were seeded at density of 2500 cells/cm^2^ in 24-well plates (Costar, Acton, MA) and incubated in either normoxia or hypoxia. After 24 h, growth media was replaced by media containing TEA in concentrations ranging from 0.2 to 100 mM. After 4 days in culture, cells were lysed with 0.02% SDS in DNAse free water for 20 min and the number of cells in the lysate was determined by using a cell quantification kit according to the manufacturer’s instructions (CyQuant, Molecular Probes, Eugene, OR) Fluorescence intensity was measured with excitation at 485 nm and emission at 535 nm on a microplate reader (Wallac Victor 2, Perkin Elmer, Hvidovre, Denmark). The experiments were performed in quadruplicates in separate experiments for all three ASC cultures.

### Electrophysiological measurements

For the electrophysiological measurements, ASCs were cultured at a density of 5000 cells/cm^2^ in 12-well culture plates (Costar, Acton, MA). After 4 days in culture, cells were detached from the surface with a mixture containing 0.01% ethylene diamine tetraacetic acid (EDTA, Titriplex III, Merck Millipore) and 0.125% trypsin (Invitrogen) and suspended in growth medium. Aliquots of the cell suspension were transferred to untreated glass coverslips and cells were allowed to settle for 30 to 45 min at room temperature. For the cells cultured in hypoxia, the settling process was performed in the hypoxic workstation. Coverslips were mounted in a custom-built acrylic chamber on an inverted microscope (BX51WI, Olympus, Japan) and perfused at 1 ml/min with a Tyrode’s solution at room temperature. The Tyrode’s solution contained (in mM) 146 NaCl, 5 KCl, 1 MgCl_2_, 2 CaCl_2_, 10 HEPES, and 11 glucose, adjusted to pH = 7.4. To assess the effect of the K^+^ blocker TEA on whole-cell currents, 20 mM TEA was included in the perfusion solution. For the hypoxic experiments, the perfusion solution was equilibrated with N_2_, resulting in an oxygen tension of 5±1% in the chamber, as monitored with an oxygen meter (OX10, Unisense, Arhus, Denmark). Patch micropipettes were fabricated using borosilicate glass (OD 1.5 mm, Sutter Instruments Co., Novato, CA) in a programmable Flaming/Brown micropipette puller (P-97, Sutter). After fire polishing, micropipettes had a tip resistance between 4 to 5 MΩ when filled with internal solution. Internal solution comprised (in mM) 10 NaCl, 1 MgCl_2_, 10 HEPES, 5 EGTA, and 120 L-Aspartic acid K^+^ salt, adjusted to pH = 7.25. Whole-cell recordings were performed using a patch-clamp amplifier (Axopatch 200B, Axon Instruments/Molecular Devices Corp., Union City, CA). Membrane currents were filtered at 1 kHz using an Axopatch 200B internal low-pass filter and acquired at 5 kHz using a PC equipped with a digitizer board (PCI-MIO-E4, National Instruments, USA) and a data acquisition software (AxographX, Axograph Scientific, New South Wales, Australia). Capacitive and leakage currents were filtered on line by means of the p/n leak subtraction routine in AxographX. Membrane currents were elicited by 300-ms voltage pulses from a holding potential of −60 mV, over the range of −60 mV to 80 mV, in 20 mV increments. To construct the current-voltage curves, the current values were calculated by averaging the measurements at the end of the plateau phase (150 to 300 ms).

Steady state currents evoked by the voltage steps were converted into conductance by using the equation *G = I/(V−V_K_)*, where *V* is the membrane potential and *V_K_* is the reversal potential (calculated as −80 mV for the conditions in this work). The normalized conductance (*G/G_max_*) curves were fitted with a Boltzmann equation *G/G_max_ = (1+ exp (V−V_0.5_)/k)^−1^*, where *V_0.5_* represents the potential in which activation reaches 50% of its maximum and *k* is the slope factor, which describes the steepness of the curve.

### RNA isolation and cDNA synthesis

Cells were seeded in 12-well plates (Costar) at different densities. For the time lapse experiment, the cell concentrations were as follows (in number of cells per 3.8 cm^2^ well): 60,000 for day 0, 50,000 for day 1, 30,000 for day 4, 20,000 for day 7, 5,000 for day 10, and 2,000 for day 13. For the experiment in which gene expression was analyzed only on day 4, 30,000 cells/well were seeded. The cells were cultured in either normoxia or hypoxia, and lysed for RNA extraction on the designated days. The experiments were performed twice and in duplicate on all three cell cultures. Total RNA was isolated using the Aurum Total RNA purification kit (Bio-Rad, Copenhagen, Denmark) according to the manufacturer’s instructions. The concentration and quality of the RNA was assessed spectrophotometrically (ND-1000, NanoDrop, Thermo Science, Wilmington, DE). cDNA was made from equal amounts of RNA input using the iScript cDNA synthesis kit (Bio-Rad). To generate positive controls, cDNA was made from RNA from human heart and brain.

### Semiquantitative real-time RT-PCR

The primer sequences (Table 1) were designed using the open source software Primer3 [27] to provide PCR product sizes between 50–110 bp. All primers were produced by DNA Technology (Arhus, Denmark). Each reaction contained 12.5 µL of SYBR Green Supermix (Bio-Rad), 1 µL of forward and reverse primers (5 pmol), 1.5 µL water, and 10 µL cDNA. Each sample was analyzed in duplicates in a single-colour real-time PCR detection system (MyIQ, Bio-Rad) using a two-step amplification cycle. The thermocycling protocol consisted of an initial step of 3 min at 95°C, followed by 40 cycles of 10 s at 95°C for denaturation and 30 seconds at 60/58°C for annealing and elongation. Product specificity was verified by melting curve analysis. Peptidylprolyl isomerase A (PPIA) and tyrosine 3-tryptophan 5-monooxygenase activation protein (YWHAZ) were also amplified as control genes and used for normalization [28]. The relative gene expression for each gene was calculated on the basis of a standard curve that was derived by pooling all the cDNA samples including positive controls (from heart and brain) and making 4-fold dilutions. In each assay, a negative control without cDNA was included. The experiment was conducted twice on each cell culture, using independent cell samples.

**Table 1:** List of genes, primer sequences, and annealing temperatures (AT) used in this study.

Genesymbol	Gene description	Primer base sequence (5′-3′)		AT (°C)
		Forward	Reverse	
**alpha1c**	Voltage-gated L type Ca^2+^ channel,alpha 1C subunit	TTT CAC CCCAATGCC TAC C	CAC TAA AAA GCC CCA CC	60
**Kv 1.1**	Voltage-gated K^+^ channel, shaker-related subfamily,member 1	AAA AGT GGG GAA GGG TTG G	GAA ATG AGA GAG CGA GAA TGG	60
**Kv 2.1**	Voltage-gated K^+^ channel, Shab-related subfamily,member 1	GTG AGG TTC TTT GCC TGT CC	TGA TGA AGT AGG GGA TGA TGG	60
**Kv 3.4**	Voltage-gated K^+^ channel, Shaw-related subfamily,member 4	CTG TCA TCG TCA ACA ACT TCG	GTG CTT CTT CCG TTT CTT GG	60
**Kv4.3**	Voltage-gated K^+^ channel, Shal-related subfamily,member 3	CTG TCA TCG TCA ACA ACT TCG	TTC CCT GCA ATC GTC TTA GG	60
**Kv7.3**	Voltage-gated K^+^ channel, KQT-like subfamily,member 3	GGA GAG GAG ATG AAA GAG GAG	TGA AGA AAG GAA AAG AGA CGA	60
**Kir 2.1**	Inwardly-rectifying K^+^ channel, subfamily J,member 2	GGA AGA CGA CAG TGA AAA TGG	ACT TGC CTG GTT GTG AGG G	58
**HCN2**	Hyperpolarization activated cyclic nucleotide-gatedK^+^ channel 2	GCT TCA CCA AGA TCC TCA GC	CCA GGT CAT AGG TCA TGT GG	60
**Maxi K**	Large conductance Ca^2+^-activated K^+^ channelsubfamily M	AAA ACA ACC AGG CTC TCA CC	AAA CAT CCC CAT AAC CAA CG	60
**PPIA**	Peptidylprolyl isomerase A	TCC TGG CAT CTT GTC CAT G	CCA TCC AAC CAC TCA GTC TTG	60
**YWHAZ**	Tyrosine 3/tryptophan 5-monooxygenaseactivation protein	ACT TTT GGT ACA TTG TGG CTT CAA	CCG CCA GGA CAA ACC AGT AT	60

### Statistical Analysis

The data is represented as mean ± standard error of the mean (SEM), except for the bar graphs where data is represented as mean + SEM. Paired Student’s t-test was performed to evaluate the difference between two related group means. Mann-Whitney rank sum test was performed to evaluate the difference between independent conditions that did not follow a normal distribution. One-way analysis of variance (ANOVA) was performed when comparing more than two groups of samples. The growth data was fit to a 4-parameter logistic regression model to estimate the rate of growth reduction and the half maximal inhibitory concentration (IC_50_) from the dose-response curves. Statistical calculations were performed using IBM SPSS Statistics v.19 (IBM SPSS, Chicago, IL).

## Results

### Analysis of TEA-induced growth inhibition in cells cultured at 5% oxygen

**Figure 1:**
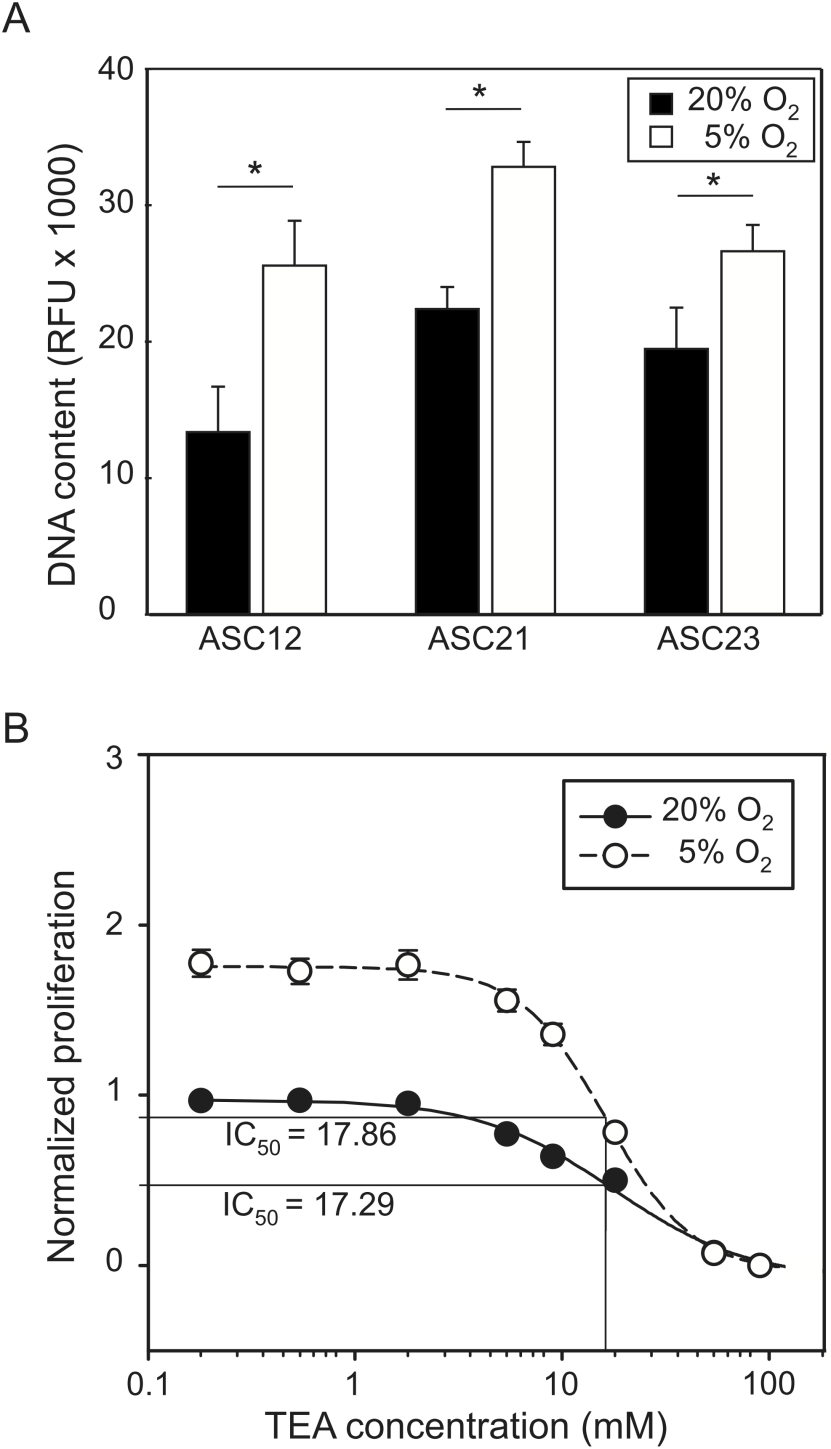
Growth and dose-dependent growth inhibition of ASCs in normoxia and hypoxia. (A) Cell numbers were greatly increased under hypoxic conditions after 4 days in culture for all three cell cultures (n = 6). Asterisk denotes statistically significant difference (p<0.01). (B) Dose-dependent growth inhibition of ASCs by TEA. The data were obtained from repeated experiments using all three cell cultures (n = 6) and was normalized to the number of cells in normoxic cultures without TEA. The half maximal inhibitory concentration (IC_50_) is indicated.

Previous studies demonstrated that blockage of K^+^ channels by TEA possess a significant anti-proliferative effect in ASCs [19]. On the other hand, it is known that culture of ASCs at 5% increases their proliferation rate [4], [5]. Therefore, the dose-dependent growth inhibition by TEA on cells cultured under low oxygen tension was investigated. As expected, all three ASC cultures exhibited an increased proliferation rate when cultured in low oxygen, which was evidenced by an average of 44% higher cell numbers after 4 days in culture (Fig. 1.A). Addition of TEA resulted in a dose-dependent growth inhibition with average half maximal inhibitory concentration (IC_50_) of 17.29 mM and 17.86 mM from ASCs cultured in normoxia and hypoxia, respectively (Fig. 1.B). The difference on the IC_50_ was, however, not statistically significant (p>0.05, n = 6).

### Outward currents recorded in ASCs cultured in normoxia and hypoxia

**Figure 2:**
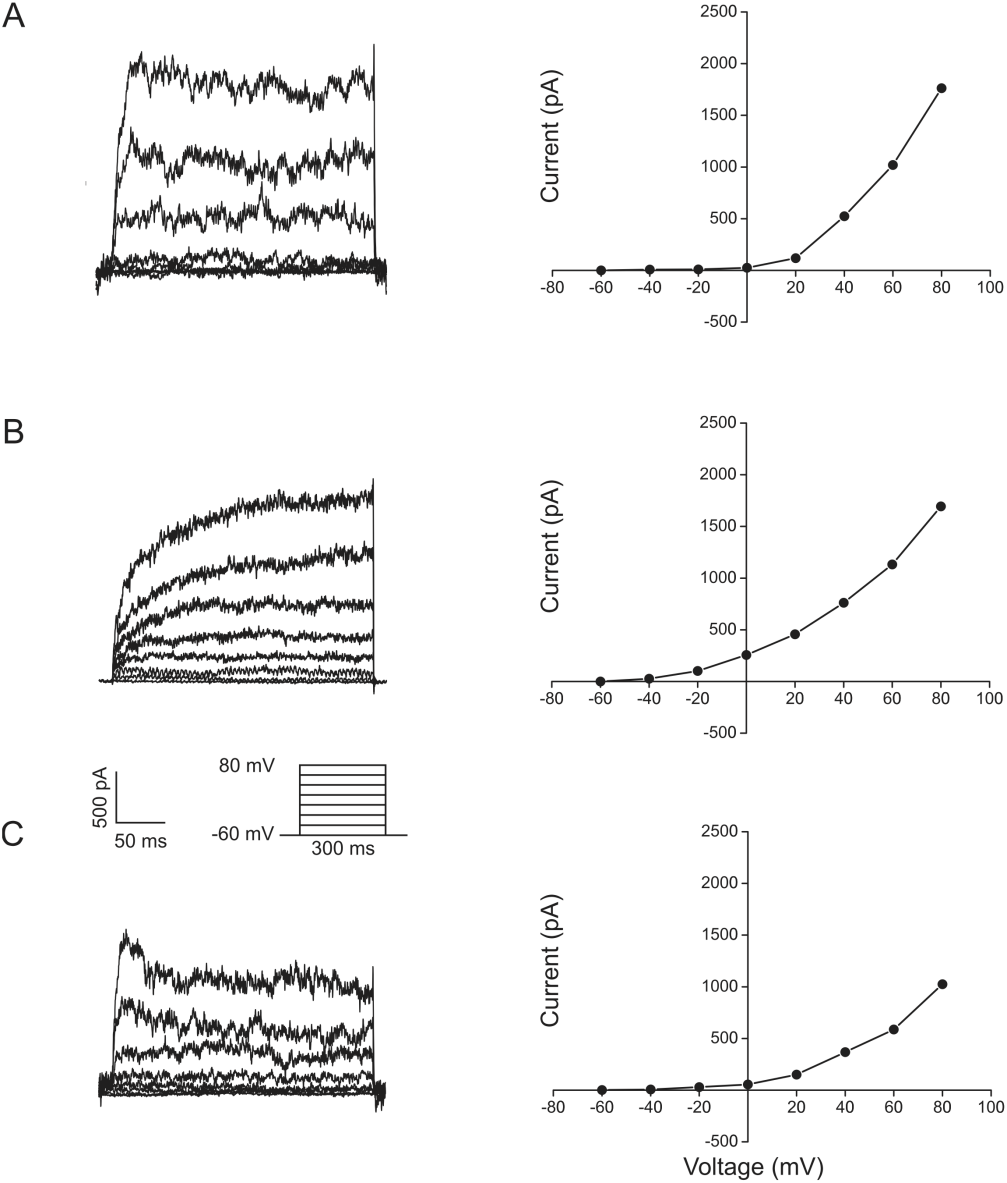
Whole-cell currents elicited in ASCs subjected to normoxic or hypoxic growth conditions. ASCs cultured in 20% and 5% oxygen tension for 4 days exhibited similar outward current patterns. Representative recordings are presented along with the respective current-voltage relationships. (A) Cell with a predominant Ca^2+^-activated K^+^ component (IK_Ca_). (B) Cell with a predominant slowly activating delayed rectifier component (IK_DR_). (C) Cell displaying a transient outward component (I_to_).

To investigate the effect of oxygen on the electrophysiological properties of the cells, whole-cell patch-clamp recordings were obtained from ASCs after 4 days of culture in either 20% or 5% oxygen. In both experimental conditions all cells displayed outward current profiles consistent with those previously described for ASCs [19], [29]. The outward K^+^ currents in ASCs are composed by a rapidly activating component with noisy oscillations like Ca^2+^-activated K^+^ currents (IK_Ca_), a slowly activating component like delayed rectifier current (IK_DR_), and a transient outward component (I_to_). The profile of the outward current trace varies from cell to cell depending on the relative contribution of each component to the total outward K^+^ current. In our experiments, the cells were classified in three groups according to their outward current profile (Fig. 2). The first group comprised cells displaying noisy currents with a fast onset and activation at near-zero or positive voltages (Fig. 2.A), which is consistent with the IK_Ca_. The second group consisted of cells displaying currents with a slower onset, activation at negative voltages and less noisy oscillations (Fig. 2.B), which is associated with the IK_DR_ component. The last group is essentially similar to the first one, with the evidence of a transient component (Fig. 2.C) attributable to the I_to_. The numbers of cells displaying patterns 1, 2, and 3 in the normoxic group were 24 (45%), 25 (47%), and 4 (8%), while in the hypoxic group were 19 (40%), 24 (51%), and 4 cells (9%), respectively.

### Effect of hypoxia on the voltage-dependence and activation kinetics of outward currents

**Figure 3:**
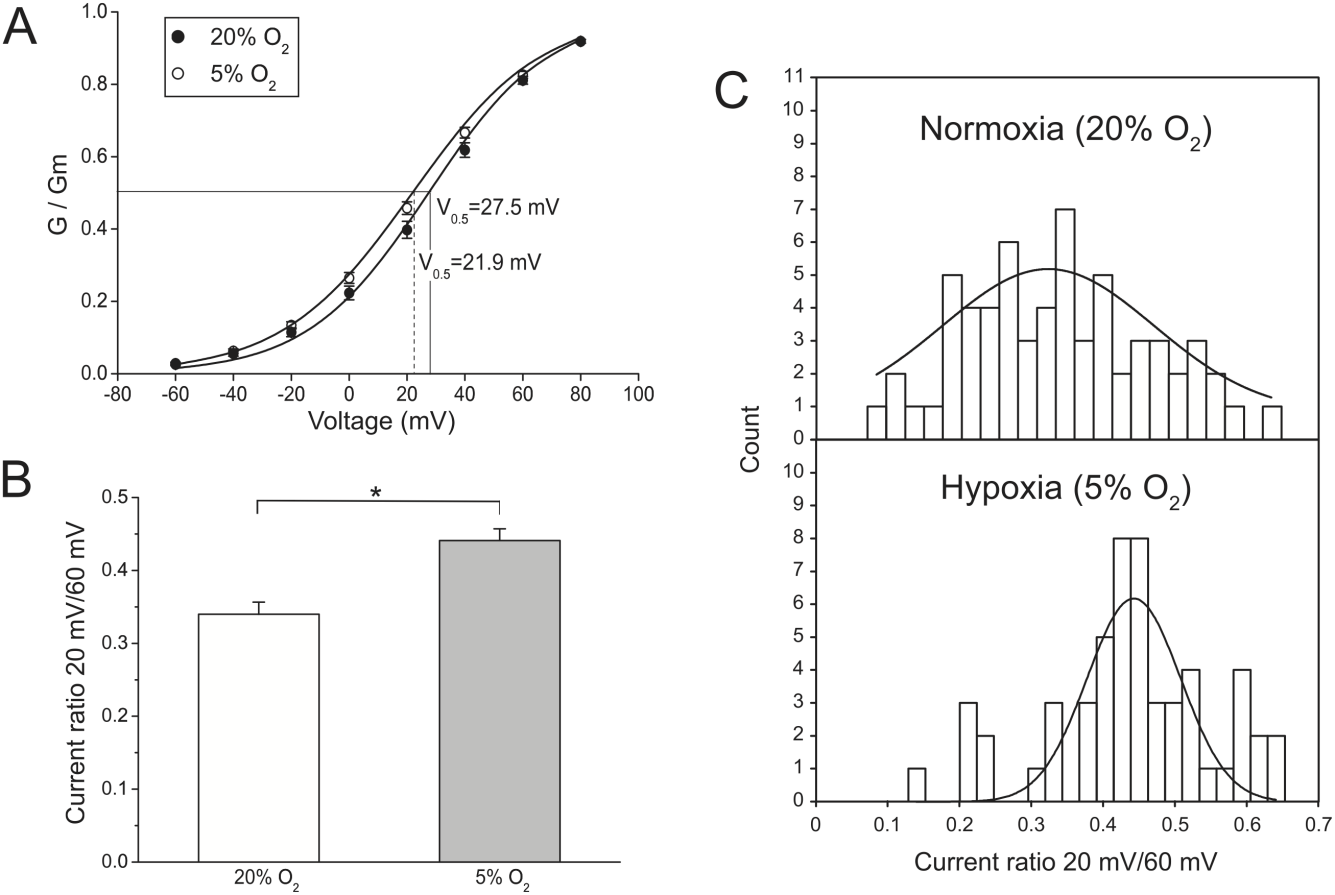
Effect of hypoxia on the activation kinetics and voltage dependence of activation of outward currents. (A) The mean normalized conductance data are presented for normoxic (n = 53) and hypoxic cultures (n = 47). To obtain the steady-state activation curves, the data were fitted using the Boltzmann sigmoid equation. Hypoxic culture shifted the steady-state activation to the left, indicating that lower voltages were required to open the channels, but the slope factor *k* was not changed. The potential where activation reached 50% of its maximum (*V_0.5_*) is indicated. (B) The graph shows the average ratio between currents at 20 mV and 60 mV for normoxic and hypoxic cultures. The asterisk (*) denotes a statistically significant difference (p<0.05). (C) Frequency distribution histograms for the 20 mV/60 mV current ratios. Data were fitted using Gaussian distribution functions.

To quantify the effects of hypoxia on the activation kinetics of the outward currents, the steady state activation curves were compared between the two groups. The Boltzmann sigmoid equation was used to obtain the parameters *V_0.5_* and *k* from each cell. While the *V_0.5_* was significantly reduced by hypoxia (21.9±1.9 mV (n = 47) vs. 27.5±1.5 mV in normoxia (n = 53), p<0.05), the slope factor *k* was not significantly changed (22.5±0.6 mV (n = 47) vs. 21.0±0.7 mV in normoxia (n = 53), p>0.05). Figure 3.A displays the steady state activation curves, in which is possible to observe that hypoxia negatively shifted the voltage-dependence of activation of the outward currents, without affecting the slope factor *k*.

Previously, it has been shown that the activation threshold and steepness of the outward current-voltage relationships from MSCs could be simultaneously assessed by calculating the ratio between currents obtained at 20 mV and 60 mV [18]. Current voltage curves with a positive activation potential and a steep slope are associated with a relatively small current ratio and the other way around. Based on this approach, the average current ratios for each experimental condition were compared (Fig. 3.B and 3.C). The ratio was significantly larger for the cells cultured under hypoxic conditions, consistent with the presence of a larger proportion of cells having more negative voltage activation thresholds and less steep current voltage curves. The modification of the current ratio for cells under hypoxia becomes more evident when plotting the frequency distribution histograms, which show a much narrower distribution, with current ratios lying above 0.3 for most of the cells under hypoxia (Fig. 3.C).

### Effect of hypoxia on the TEA-mediated shift of activation threshold

**Figure 4:**
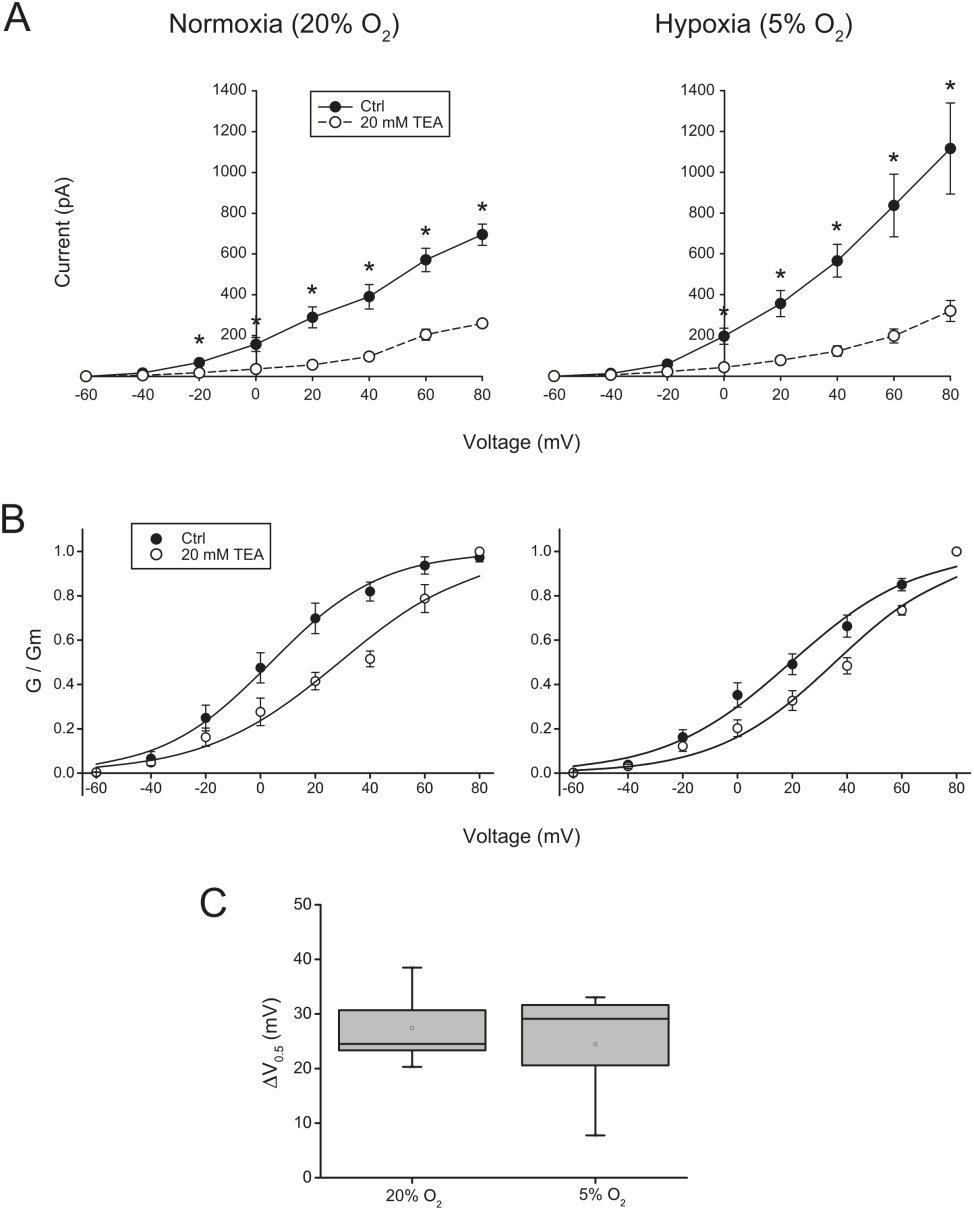
Inhibition of outward currents by TEA in ASCs cultured in normoxia and hypoxia. (A) Reduction in the steady-state current values as a result of 10 min perfusion with 20 mM TEA. Asterisks (*) denote a statistically significant difference (p<0.05) between the control and TEA-treated cells (n = 6). (B) TEA-mediated shift of the steady-state activation curves. The mean normalized conductance data was fitted using the Boltzmann sigmoid equation. (C) Boxplot (middle line: median; box: upper and lower quartile; bars: minimum and maximum values) of the change in *V_0.5_* after addition of TEA. No statistical significant differences were found (Mann-Whitney U test).

Most cells displayed outward currents consistent with the presence of IK_DR_ or IK_Ca_ components (Fig. 2). K^+^ channels underlying these currents are non-selectively blocked by mM concentrations of TEA, which shifts the voltage of activation to more positive potentials. Given the role of these channels in the regulation of MSCs proliferation, the effect of hypoxia on the TEA-mediated current reduction and voltage of activation shift was studied. Whole cell recordings were performed on ASCs that were exposed to 20 mM of TEA in the perfusion solution. As expected, TEA significantly inhibited the K^+^ outward currents in both normoxia and hypoxia (n = 5, p<0.05 in both conditions) (Fig. 4.A). The activation kinetics curves were shifted by TEA, leading to significant differences in the voltage activation thresholds (Fig. 4.B). The values for Δ*V_0.5_* in normoxia and hypoxia were −19.6±8.0 mV (n = 5, p<0.05) and −12.4±3.7 mV (n = 5, p<0.05). However, comparison of these differences between the two groups did not produce statistically significant results (Fig. 4.C), indicating that hypoxia does not significantly affect the TEA-mediated shift of voltage activation threshold of the K^+^ outward currents.

### Transcriptional control of ion channel subunits in ASCs exposed to hypoxic growth conditions

**Figure 5:**
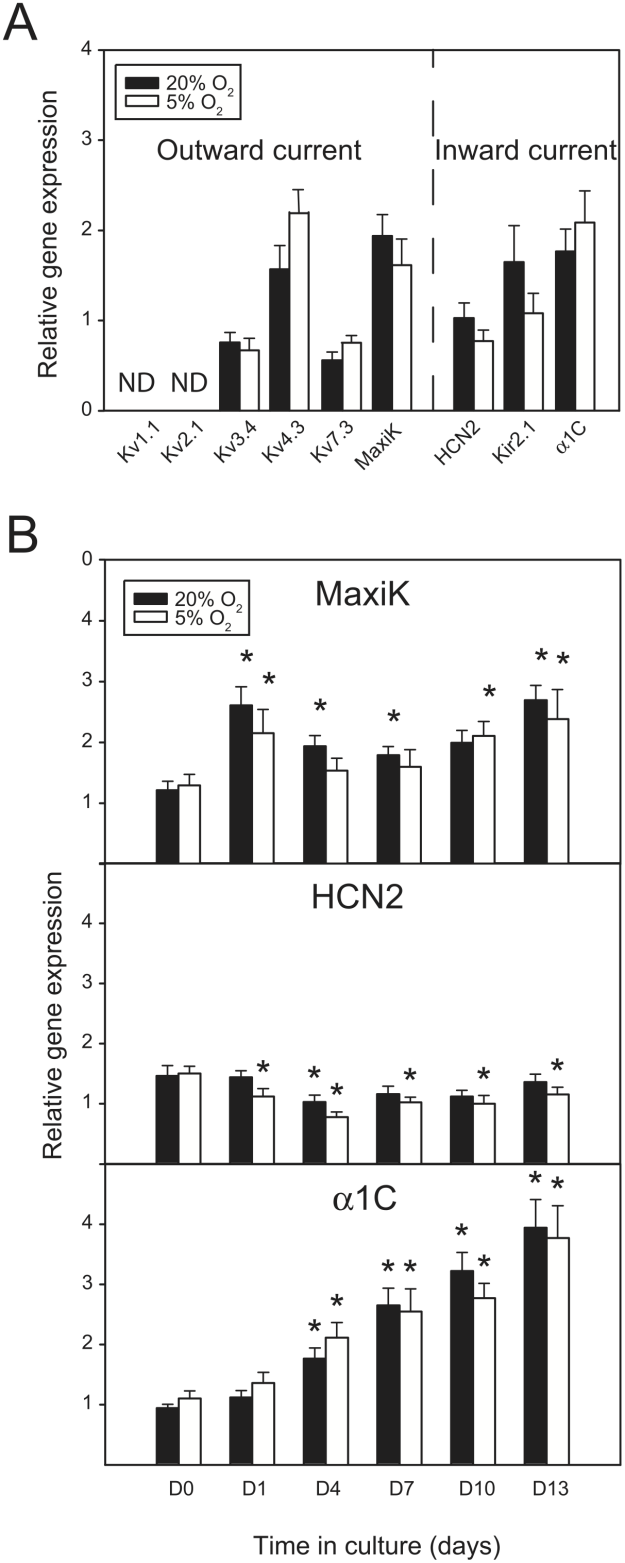
Ion channel gene expression in response to hypoxia. (A) Transcriptional expression of selected outward and inward ion channel genes (Kv1.1, Kv2.1, Kv3.4, Kv4.3, Kv7.3, MaxiK, HCN2, Kir2.1, and α1C) was assessed after 4 days of culture in standard normoxic conditions and in hypoxia. (B) Expression of MaxiK, HCN2 and α1C, genes during 13 days of culture in 20% and 5% oxygen concentration. The data were obtained from repeated experiments using all three cell cultures (n = 6). The asterisks (*) denote a statistically significant difference (p<0.05) with respect to D0.

To investigate the molecular correlate for the observed effects of hypoxia at functional level, the levels of transcripts for ion channel subunits in ASC cultures were analyzed by quantitative RT-PCR. Samples from human cardiac and brain cells were used as a positive control. The expression of Kv1.1, Kv2.1, Kv3.4, Kv4.3, Kv7.3, MaxiK, HCN2, Kir2.1, and α1C genes was assessed in cultures after four days of expansion at 20% or 5% oxygen concentrations (Fig. 5.A). No significant difference between normoxic and hypoxic condition was found in Kv3.4, Kv4.3, Kv7.3, MaxiK, HCN2, Kir2.1, and α1C, whereas the Kv1.1 and Kv2.1 subunits were not detected. Based on positive signals from the standard curves that included cDNA from heart and brain, it was confirmed that the lack of detection of Kv1.1 and Kv2.1 in ASCs was indeed due to lack of expression and not methodological errors. The expression profiles of MaxiK, HCN2 and α1C subunits, which are all implicated in regulation of cell proliferation, were further studied over a period of 13 days (Fig. 5.B). Statistical analysis showed no differences in the gene expression levels between the cells cultured in normoxia and hypoxia. However, a time-dependent modulation of transcriptional activation was observed, with patterns featuring upregulation in the case of α1C and MaxiK, and downregulation in HCN2. These changes were statistically significant for at least one time point for both conditions.

## Discussion

In this study, we have for the first time assessed the electrophysiological profile of ASCs cultured in moderate hypoxia. ASCs displayed outward currents comprising a rapidly activating component (IK_Ca_), a slowly activating component (IK_DR_), and, in a minor proportion, a transient component (I_to_). As shown by the TEA-mediated growth inhibition assay, K^+^ channels underlying the main components of the outward current (IK_DR_ and IK_Ca_) play a key role on the proliferation of ASCs. These results are in agreement with the findings of Bai et al., who showed that TEA given to the ASC cultures in concentrations up to 120 mM significantly reduced the proliferation of ASCs in a dose-dependent manner [19]. In their work, it was shown that the cytotoxicity of TEA is negligible at concentrations at which the blocker effectively causes an inhibitory effect on cell proliferation by blocking the K^+^ channels. Our results are also consistent with studies performed in BMSCs, in which cell proliferation was linked to Kv10.1 channels (associated with IK_DR_) and MaxiK channels (associated with IK_Ca_) [22]. Although cells cultured under hypoxic environment displayed a significantly larger proliferation rate, their sensitivity towards the blocker was not altered, as evidenced by a dose-response curve with similar parameters. Also, when the K^+^ outward currents of ASCs cultured in hypoxia were blocked by 20 mM TEA, the shift in the activation threshold was not significantly different from that of the control condition. Furthermore, the transcriptional activity of subunits known to be sensitive to TEA was either below detection threshold (for Kv1.1 and Kv2.1) or unaffected by hypoxic culture (Kv3.4 and MaxiK). This is particularly intriguing in light of the major accepted mechanism behind oxygen-sensitive K^+^ channels, in which the closed state is favored by low oxygen tension, leading to membrane depolarization and subsequent activation of voltage dependent Ca^2+^ channels [14], [15], [30]. For instance, in smooth muscle cells, TEA-sensitive K+ channels respond to oxygen changes by affecting Ca^2+^ regulation, which in turn has an effect on cell contractility and proliferation [12]. Thus, our data clearly indicates that K^+^ channels sensitive to TEA do not mediate the increased proliferation of human ASCs under hypoxia. Other oxygen-sensitive K^+^ subunits, which are relatively insensitive to TEA, comprise the Kv4.x channels mediating the transient outward currents (I_to_) [31]. Studies have shown that the I_to_ blocker 4-aminopyridine significantly reduces ASCs proliferation in a dose-dependent manner [19]. However, I_to_ is present in a relatively small population of the ASCs and the transcriptional activity of the subunit underlying this current (Kv4.3) was unaffected by hypoxic culture.

Patch-clamp measurements were performed to examine whether the whole-cell currents in ASCs were altered due to culture in hypoxia. Qualitative inspection of the current recordings indicated that the percentage of cells displaying one of the three outward current patterns remained essentially unchanged in hypoxia. However, quantitative analysis of the activation kinetics of the outward currents revealed a significant effect of the hypoxic culture. Interestingly, the ASCs cultured in hypoxia displayed a negative shift in the voltage dependence of activation of the outwards currents. Moreover, when comparing the profiles of current-voltage relationships, it was shown that hypoxic cultures had a larger proportion of cells possessing outward currents with more negative voltage activation thresholds and less steep current voltage curves. This particular profile seems to be either induced or selectively favored by the hypoxic culture. Although the results of this paper do not allow in concluding which is the prevalent mechanism, other studies provided evidence supporting that hypoxic culture may in fact select for particular subpopulations in a heterogeneous population of stem cells [32], [33].

The shift to a lower activation threshold in hypoxic cultures may have a direct consequence on the resting membrane potential, which is one of the key regulators of proliferation in a number of cell types [34], [35]. It is generally known that IK_DR_ and IK_Ca_ channels regulate the resting membrane potential and play a central role in controlling the progression through the cell cycle [21], [36]. In particular, when electrophysiological properties of human ASCs from visceral and subcutaneous sources have been compared, it was shown that outward currents of subcutaneous ASCs display more negative voltage thresholds, which was associated with more hyperpolarized resting membrane potentials [29]. Also, it has been observed that membrane currents with low activation threshold consistently shifted the membrane potential of BMSCs toward more negative values [18]. Although we have not systematically investigated the effect of hypoxic conditions on the membrane potential in our cells, a possible explanation for an increased cell growth under hypoxic conditions might be a negative shift in the membrane potential. The observation that the Ca^2+^ ionophore ionomycin induces membrane hyperpolarization and stimulates proliferation of BMSCs, seems to support this idea [21].

In regards to the transcriptional analysis of ion channel subunits by semiquantitative RT-PCR, we confirmed the observation by other groups that Kv4.3, Kv7.3, MaxiK, HCN2, Kir2.1, and α1C, are expressed in ASCs [19]. However, we could not detect the expression of subunits Kv1.1 and Kv2.1. Interestingly, Kv1.1 and Kv2.1 are known TEA- and hypoxia-sensitive Kv subunits, which play a key role in conferring the oxygen sensitivity to excitable cells [8], [12], [31]. Furthermore, when analyzing a range of outward K^+^ channels that have been previously shown to be transcriptionally regulated by hypoxia in other cell types, we did not detect any significant changes in the expression patterns [31], [37], [38]. Taken together, these results support the concept that a different mechanism of oxygen sensitivity may exist in ASCs. However, the analysis of the transcription of additional ion channels such as HCN2, Kir2.1, and α1C did not immediately reveal any probable candidate responsible for the increased proliferation of the ASCs. These subunits have been associated to K^+^ and Ca^2+^ inward currents recorded in a relatively small population of MSCs [19], [20]. However, we did not come across any cell exhibiting inward currents in our patch-clamp experiments, given that activation of these channels should be negligible at voltages higher than −60 mV [29].

In conclusion, we have shown that oxygen modulates K^+^ outward currents in ASCs. When cells are cultured under moderate hypoxic conditions, the slope of the current-voltage curve is reduced and the voltage activation threshold is shifted toward more negative values. This altered profile may have a direct consequence on the resting membrane potential, as membrane hyperpolarization stimulates cell proliferation. Although TEA-sensitive K^+^ channels have been previously implicated in the altered cell behavior under hypoxic culture, we could not find evidence supporting the involvement of TEA-sensitive subunits in the increased proliferative activity displayed by ASCs. The association between hypoxia and expression profile of other ion channels that are either involved in cell cycle control or regulated at transcriptional level by oxygen in various cell types, could not be found in the present work. The identification of additional subunits and the development of more precise functional assays using specific blockers are required to fully understand the mechanism by which hypoxia modulates the electrophysiological properties of ASCs. In summary, this study represents an important step towards understanding the impact of moderate hypoxia on the electrophysiological properties of ASCs and provides new insight that may be of use in future studies investigating the effects of hypoxic preconditioning on ASCs.
